# *Bartonella quintana* Characteristics and Clinical Management

**DOI:** 10.3201/eid1202.050874

**Published:** 2006-02

**Authors:** Cédric Foucault, Philippe Brouqui, Didier Raoult

**Affiliations:** * Université de la Méditerranée, Marseille, France

**Keywords:** Bartonella quintana, trench fever, homeless, endocarditis, bacteremia, bacillary angiomatosis, diagnosis, treatment

## Abstract

*Bartonella quintana*, a pathogen that is restricted to human hosts and louse vectors, was first characterized as the agent of trench fever. The disease was described in 1915 on the basis of natural and experimental infections in soldiers. It is now recognized as a reemerging pathogen among homeless populations in cities in the United States and Europe and is responsible for a wide spectrum of conditions, including chronic bacteremia, endocarditis, and bacillary angiomatosis. Diagnosis is based on serologic analysis, culture, and molecular biology. Recent characterization of its genome allowed the development of modern diagnosis and typing methods. Guidelines for the treatment of *B. quintana* infections are presented.

## Abstract 2

The pathogen is reemerging in the United States and Europe and is responsible for a number of clinical conditions.

Trench fever, the first clinical manifestation attributed to *Bartonella quintana*, affected an estimated >1 million people during World War I (*1**,**2*). The name "trench fever" was mentioned for the first time in 1915 (*3**,**4*). In 1916, McNee et al. described 2 types of the disease (*5*). The first was characterized by a sudden onset of headache, dizziness, pain in the shins, and elevated temperature (39°C–40°C). Between days 3 and 7, temperature would suddenly drop to normal or subnormal. Thereafter, temperature rose sharply before falling again. The second manifestation of the disease was characterized by a shorter initial period and frequent relapses. In 1919, 200 consecutive cases were recorded by Byam et al., and transmission by human body lice was demonstrated, but the nature of the trench fever agent was still unknown (*4*).

Trench fever was precisely described based on experimental infections in volunteer soldiers (*4*). The first experiments consisted of transmitting whole blood from typical cases to volunteers, which reproduced natural infection. Byam confirmed in 1919 the others' work, showing that "rickettsia bodies" were present in lice, their excreta, and their guts when they were collected from trench fever patients. In 1949, Kostrzewski precisely described trench fever after an accidental epidemic spread among louse-feeders in laboratories that produced typhus vaccine (*6*). Of 104 persons who worked with lice, 90 contracted symptomatic trench fever, and 5 were asymptomatic carriers. Three different courses of trench fever were described by Kostrzewski: the classic relapsing form associated with shin pain, headaches, and dizziness; the typhoidal form characterized by a prolonged fever, splenomegaly, and rash; and the abortive form, characterized by a brief, less intense course.

After World War I, the incidence of trench fever decreased dramatically, but during World War II, epidemics were again reported (*6*). More recently, reports have indicated the reemergence of *B. quintana* infections among the homeless population in cities in both Europe and the United States (*7**,**8*). Major predisposing factors for new *B. quintana* infections include poor living conditions and chronic alcoholism (*8*). Epidemics of trench fever were also recently reported in particular conditions, such as in refugee camps in Burundi in 1997, where pediculosis was prevalent (*1*).

## The Bacterium

### Taxonomy

When trench fever was first described in 1915, its etiologic agent was called *Rickettsia quintana* or *R. volhynica* (*6*). At the same time, other names were also proposed, i.e., *R. pediculi*, *R. weigli*, and *R. rocha-limae* (*1*). The 1984 edition of Bergey's Manual of Systematic Bacteriology combines *Rickettsiaceae*, *Bartonellaceae*, and *Anaplasmataceae* families into the *Rickettsiales* order. *R. quintana* was classified in the genus *Rochalimaea*, tribe *Rickettsiae*, family *Rickettsiaceae*. In 1993, Brenner et al. proposed unifying the genera *Bartonella* and *Rochalimaea* (*9*). They also proposed removing the unified genus *Bartonella* from the *Rickettsiales* order. The new unified genus thus contained 5 species: *B. bacilliformis*, *B. quintana*, *B. vinsonii*, *B. henselae*, and *B. elizabethae*. In 1995, Birtles et al. proposed unifying the *Bartonella* and *Grahamella* genera (*10*), and the 2 established *Grahamella* species, *Grahamella talpae* and *G. peromysci*, were renamed as *Bartonella* species. Birtles et al. also described 3 new species within *Bartonella*: *B. grahami*, *B. taylorii*, and *B. doshiae* (*3**,**10*).

### Characteristics

*B. quintana* is a facultative, intracellular, gram-negative rod belonging to the α2 subgroup of proteobacteria (*3*). It is a short rod, 0.3–0.5 μm wide and 1–1.7 μm long. Catalase and oxidase reactions are negative. The bacterium can be grown on axenic media and cocultivated in cell culture (*3**,**11*). When grown on blood agar, rough colonies embedded in the agar are obtained after 12 to 14 days, but prolonged incubation may be necessary, up to 45 days for primary isolation. Subcultures reduce the time to obtain colonies to only 3–5 days (*11*). Humans are the reservoir of the bacterium (*12*), and the human body louse, *Pediculus humanus corporis*, is its usual vector (*1*). *B. quintana* is located in erythrocytes during asymptomatic bacteremia (*13*) and has been observed in erythroblasts in bone marrow in bacteremic patients (*14*). The bacterium has a tropism for endothelial cells, leading to angioproliferative lesions, as observed in bacillary angiomatosis (*15*).

### Genome

The 1.6-Mb genome of *B. quintana* has recently been sequenced and was found to be a derivate of the larger 1.9-Mb genome of *B. henselae*; the main difference between the species is the absence of genomic islands in *B. quintana* (*16*). Both *B. quintana* and *B. henselae* genomes are shortened versions of chromosome I from *Brucella melitensis*, a phylogenetically highly related bacterium (*16*). The comparison of *B. henselae* and *B. quintana* genome and the specialization of the latter to its human reservoir and louse vector suggest that use of host-restricted vectors is associated with accelerated rates of genome degradation (*16*).

## Epidemiologic Features and Natural History

### Transmission

**Figure 1:**
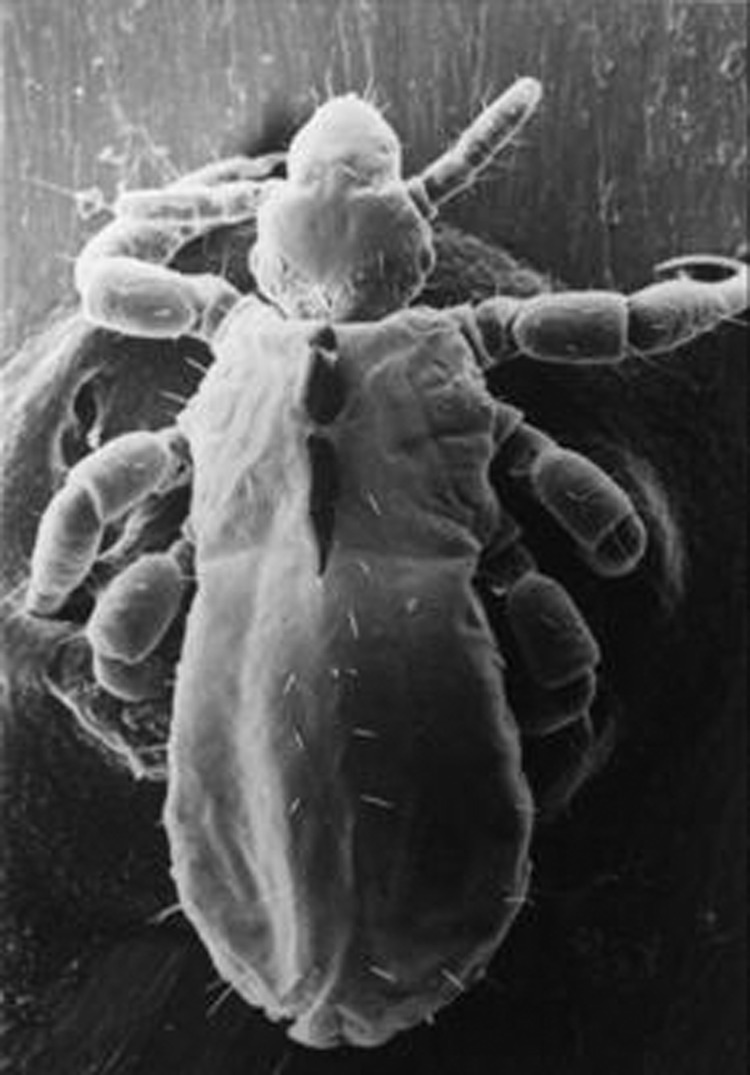
*Pediculus humanus corporis*, the human body louse, viewed with electron microscope at magnification ×120.

**Figure 2:**
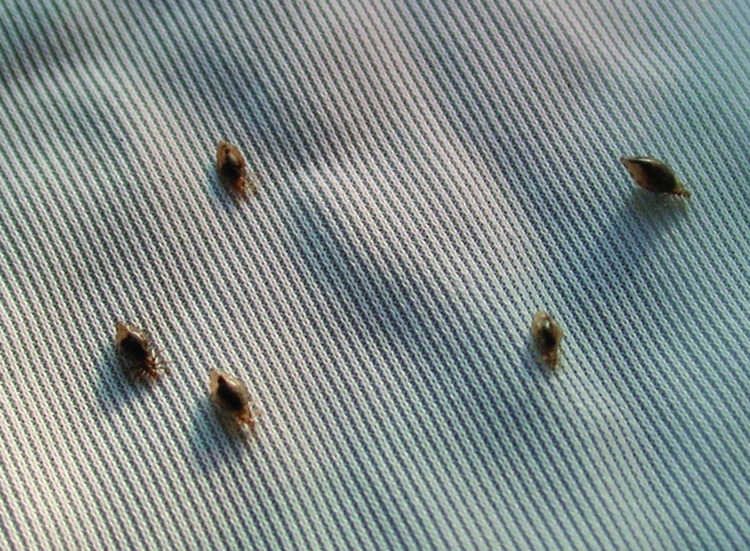
Human body lice in clothes.

**Figure 3:**
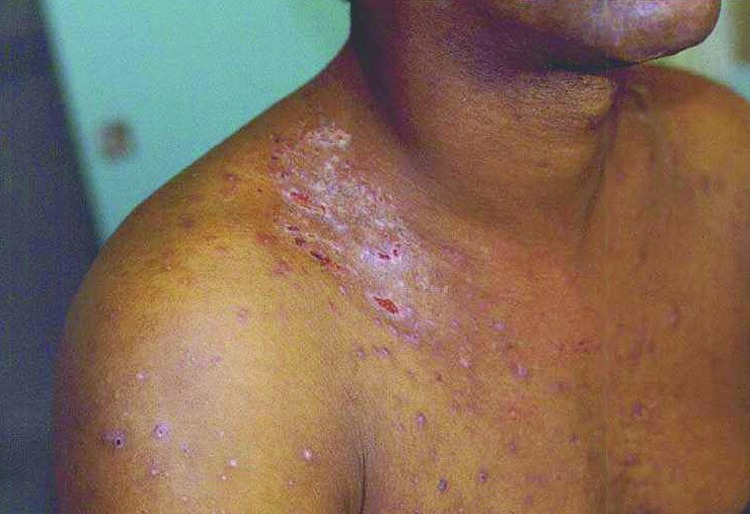
Lesions from scratching induced by body lice infestation.

*B. quintana* is transmitted by the human body louse, *P. humanus corporis* (Figure 1), which lives in clothes (Figure 2) and is associated with poverty, lack of hygiene, and cold weather. In our cohort of 930 homeless persons, lice infestation was present in 22% and was associated with hypereosinophilia (*17*). Pediculosis (lice infestation) is transmitted by contact with clothes or bedding, is prevalent in the homeless population. *B. quintana* multiplies in the louse's intestine and is transmitted to humans by feces through altered skin (*1*). Body lice usually feed 5 times a day and inject their bites with biological proteins, including an anesthetic that provokes an allergic reaction and leads to pruritus and scratching (Figure 3), which facilitates the fecal transmission of *B. quintana*, and persistent *B. quintana* bacteremia facilitates its spread by lice (*12*). Body lice are probably not the only vectors of *B. quintana*. The bacterium was recently detected in cat fleas (*18*) and in cat dental pulp (*19*), which suggests bacteremia in cats, and has been isolated in a patient who owned a cat and sought treatment for chronic adenopathy (*20*). These data suggest a transmission mode similar to that observed for *B. henselae* in cat-scratch disease.

### Natural History of *B. quintana* Infection

After trench fever (which corresponds to the primary infection) resolves, chronic bacteremia will develop in some patients (*7*). Overproduction of interleukin-10 could be partially responsible for persistence of bacteremia. The link between chronic bacteremia and *B. quintana* endocarditis has not been clearly shown, but it likely exists. Chronic asymptomatic bacteremia in humans indicates that they may be the natural reservoir of *B. quintana* (*12*), as this condition occurs with other *Bartonella* species in their reservoirs, i.e., *B. henselae* in cats, *B. alsatica* in rabbits, and *B. tribocorum* in rats. However, other reservoirs should be investigated for *B. quintana* because the bacterium has been detected in cat fleas (*18*), cat teeth (*19*), and monkey fleas (unpub. data). Recently, an animal model of *B. quintana* infection in rhesus macaques has been developed to reproduce the prolonged bacteremia that is observed in humans (*21*).

### Surveys in Homeless Shelters

A substantial seroprevalence of *B. quintana* has been reported in France (*7*) and the United States (*22*). An epidemiologic survey conducted in emergency rooms of the University Hospital in Marseilles, France, showed that 30% of 71 tested homeless persons had antibody titers against *B. quintana* and that 14% were bacteremic (*7*). We found that 50 (5.4%) of 930 nonhospitalized, homeless persons tested during 4 years (2000–2003) were bacteremic (*17*).

## Clinical Manifestations

### Trench Fever

Trench fever is characterized by attacks of fever that last 1–3 days; are associated with headache, shin pain, and dizziness; and recur every 4–6 days (*2**,**23*), although each succeeding attack is usually less severe. The incubation period typically varies from 15 to 25 days but may be reduced to 6 days in experimental infections (*3**,**4*). Although trench fever often results in prolonged disability, no deaths have been reported (*1**,**3*).

### Chronic Bacteremia

Persistent bacteremia has long been associated with *B. quintana* infection (*2**,**4*). Kostrzewski showed that *B. quintana* was present in the blood of trench fever patients up to 8 years after initial infection (*6*). More recently, asymptomatic and prolonged bacteremia was confirmed in 16 of 42 patients with positive blood cultures (*12*). Chronic bacteremia persisted for 78 weeks in 1 of those patients, for 53 and 17 weeks in 2 other patients, and for 1 to 8 weeks in the remaining 13. Intermittent bacteremia was also observed over periods of 4 to 58 weeks.

### Endocarditis

Cases and series of *Bartonella* endocarditis have been widely reported (*2**,**24*), including a report of 48 cases, 38 of *B. quintana* infection, and 10 of *B. henselae* infection (*24*). Patients appeared to have chronic, blood culture–negative endocarditis; fever was usually present (90%), a vegetation was usually observed on echocardiograph (90%), and >90% of patients required valvular surgery. *B. quintana* endocarditis mostly develops in persons without any previous valvular injuries; known risk factors are alcoholism, homelessness, and body lice infestation. *B. henselae* endocarditis patients frequently have a previous valvulopathy, and disease is associated with cat bites or scratches and cat flea exposure (*24*). Treatment and outcome of *Bartonella* endocarditis were examined on the basis of 101 cases (*25*). Patients who received an aminoglycoside were more likely to fully recover, and those treated with aminoglycosides for >14 days were more likely to survive than those treated for a shorter duration. Of the 101 patients with *Bartonella* endocarditis, 12 (11.9%) died despite antimicrobial drug therapy and valvular surgery, 10 of acute heart failure and 2 of multiorgan failure (*25*).

### Bacillary Angiomatosis

Bacillary angiomatosis was first described early in the HIV epidemic (*2**,**26*). It is a proliferative vascular disease recognized in both immunocompetent and immunodeficient patients (mostly HIV-infected persons) (*3**,**27*). *B. quintana* and *B. henselae* are the 2 etiologic agents (*26*). Various organs may be affected, including the liver, spleen, bone marrow, and lymph nodes, but the skin is most often involved (*3*). Cutaneous lesions may be solitary or multiple and may bleed profusely when punctured. They may be superficial, dermal, or subcutaneous. Superficial lesions may be red, purple, or colorless. Deep lesions are not usually colored and are either mobile or fixed to underlying structures. The oral, anal, and gastrointestinal mucosa may also be involved (*3*). Molecular epidemiologic features of *Bartonella* infections in HIV-infected patients with bacillary angiomatosis were investigated (*26*); bone lesions and subcutaneous masses were associated with *B. quintana*, whereas hepatic peliosis and lymph node lesions were associated with *B. henselae*. Bacillary angiomatosis may be life-threatening in untreated patients.

### Lymphadenopathy

*B. quintana* has been reported to cause lymphadenopathy. A 30-year-old woman with isolated chronic, afebrile, cervical, and mediastinal adenopathy was the first reported patient (*20*). Histologic examination of the cervical lymph node showed a granulomatous reaction, and *B. quintana* was isolated from blood cultures. A second case was later reported in a hemodialysis patient with Sjögren syndrome who had mediastinal lymphadenopathies and secondary pancytopenia (*28*). *B. quintana* was isolated from a bone marrow biopsy specimen, and the bacterium was identified by using molecular biologic methods. His serum showed an antibody titer of 1:50 against *B. quintana*. In 2003, a coinfection with *B. quintana* and *Mycobacterium tuberculosis* was reported in an HIV-infected patient with supraclavicular inflammatory lymphadenitis. The 2 microorganisms were isolated from lymph nodes (*29*).

## Diagnosis

### Serologic Tests

**Figure 4:**
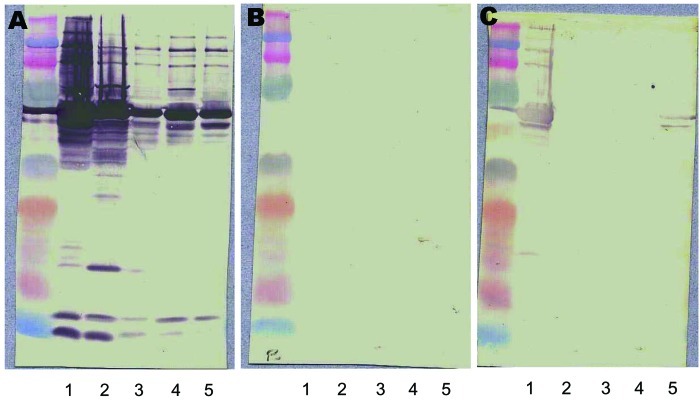
Western blot and cross-adsorption results in a patient with *Bartonella quintana* endocarditis. A) Nonadsorbed. B) Adsorbed with *B. quintana*. C) Adsorbed with *B. henselae*. Lane 1, *B. quintana*; lane 2, *B. henselae*; lane 3, *B. elizabethae*; lane 4, *B. vinsonii* subsp. *Berkhoffi*; lane 5, *B. vinsonii* subsp. *Arupensis*. Before adsorption (A), antibodies are detected against all species (1, 2, 3, 4, and 5). After adsorption with *B. quintana* antigen (B), all antibodies disappear. After adsorption with *B. henselae* antigen (C), antibodies against *B. quintana* (*1*) persist. This reaction shows *B. quintana* infection.

Serologic testing is the most widely used method to diagnose *Bartonella* infection. Indirect immunofluorescence is the reference method. Variability in antibody titers may occur when different methods of antigen preparation are used for the assay. Moreover, cross-reactions have been reported with *Coxiella burnetii* and *Chlamydia pneumoniae* (*1**,**3*). Western blot and cross-adsorption resolve this problem and can be used to determine the species involved (Figure 4) (*30*). On indirect immunofluorescence, immunoglobulin G titers >1:50 indicate *Bartonella* infection, and titers >1:800 predict endocarditis (*31*).

### Culture

*B. quintana* was first cultivated in axenic media by Vinson in the early 1960s (*32*). To date, the most widely used methods for isolation are direct plating onto solid media, blood culture in broth, and cocultivation in cell culture (*11*). From 1993 to 1998, we correlated the results of *Bartonella*-positive cultures with the type of sample, the culture procedure, and polymerase chain reaction (PCR)–based genomic detection (*11*). During this period, we received 2,043 samples of *Bartonella* species for culture and obtained 72 isolates of *B. quintana*. The most efficient culture method in patients with endocarditis was to subculture blood culture broth into shell vials. For samples from homeless patients with *B. quintana* bacteremia, subculturing blood culture broth onto agar was more efficient than direct blood plating (*11*). Lysis centrifugation has been shown to enhance the recovery of *Bartonella* species as well as sample congelation from blood. Primary isolates are typically obtained after 12–14 days, although an incubation period of up to 45 days may be necessary (*11*).

### Molecular Biology

*Bartonella* species can be detected from blood and tissues by using PCR. Various tissues may be used, including lymph node, cardiac valve, skin, and liver. Although fresh tissues are more convenient, formalin-fixed, paraffin-embedded tissues may be used for PCR-based assay as well. Using universal primers to amplify the 16S rRNA gene is not a convenient method for diagnosing *Bartonella* infection to the species level because the 16S rRNA genes of *Bartonella* species are >97.8% similar. New targets have been used to detect *Bartonella* species with a PCR-based assay (Table A1) (*33*).

### Immunohistochemistry and Immunofluorescence

**Figure 5:**
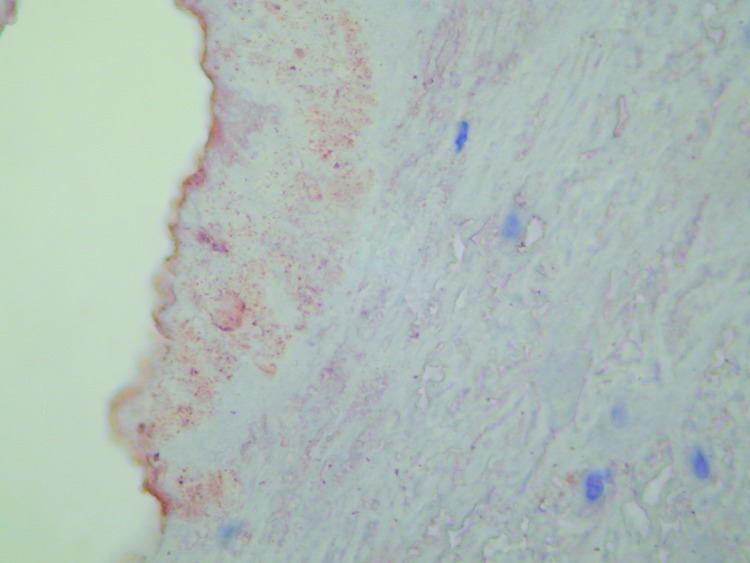
Immunohistochemical demonstration of *Bartonella* sp. in a cardiac valve of a patient with endocarditis. Magnification ×400.

Immunohistochemistry is a convenient tool for detecting *B. quintana* in tissues. Positive detection has been reported in valvular tissue (Figure 5) (*34*) and in skin biopsies of patients with bacillary angiomatosis (*35*). On immunohistochemical tests, *Bartonella* species are observed in proliferative endothelial cells localized in the upper reticular dermis in patients with bacillary angiomatosis (*35*). In patients with *Bartonella* endocarditis, clusters of bacteria, mainly in the valvular vegetation, occupy an extracellular location in the fibrin deposits (*34*).

**Figure 6:**
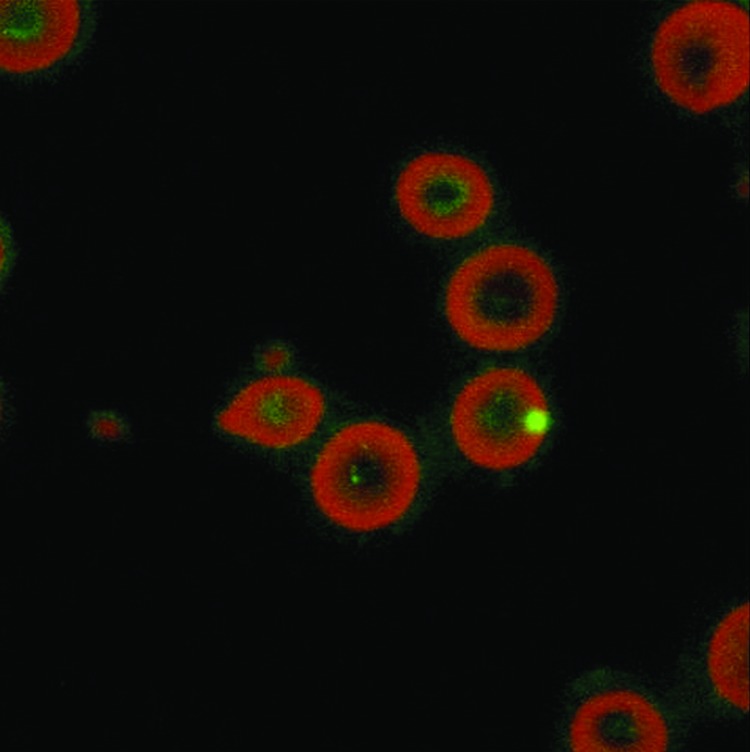
Laser confocal microscopy showing the intraerythrocytic location of *Bartonella quintana*. Magnification ×400.

*B. quintana* can be detected in erythrocytes by using immunofluorescence (*13*). The bacterium is observed in thin blood smears from fresh blood fixed with methanol and stained with mouse monoclonal antibody (*13*). The intraerythrocytic location of *B. quintana* can be confirmed by using confocal microscopy (Figure 6) (*13*).

### What Diagnostic Modality To Choose

Serologic testing and blood culture should be performed as described above when *B. quintana* infection is suspected, regardless of the clinical signs and symptoms. When endocarditis is suspected, culture, immunohistochemical tests, and PCR should be performed on the cardiac valve when available. When bacillary angiomatosis is suspected, culture, immunohistochemical tests, and PCR may be performed on a skin biopsy specimen.

## Typing

### Pulsed-field Gel Electrophoresis (PFGE)

Until recently, the only available method for typing *B. quintana* was PFGE, which allowed defining a specific pattern for each of 7 tested isolates (*36*). However, the PFGE profile does not correlate with that of the sequenced-based typing method; variability in PFGE patterns could be explained by frequent genome rearrangements in *B. quintana* (*37*). This finding was supported by the fact that the PFGE profile of 1 strain was modified after 9 subcultures while the profile from sequence-based typing remained identical (*37*), which showed that this method was unreliable in assessing epidemiologic aspects of *B. quintana*.

### Multispacer Typing

Recently, we proposed a new typing method for *B. quintana*, multispacer typing (MST) (*37*). This method, first used for *Yersinia pestis* (*38*), is based on comparing spacers, i.e., intergenic zones. Surprisingly, MST allowed us to determine only 5 different sequence types among *B. quintana* isolates. The finding of few sequence polymorphisms in the noncoding DNA of *B. quintana* agrees with findings from a previous study in which 16S–23S spacer sequencing allowed a specific sequence of each of the tested *B. henselae* isolates to be identified, while only 2 different types were identified for *B. quintana* (*36*). As *B. quintana* is a genomic derivate of *B. henselae* (*16*), its oligoclonality could have been caused by a very recent adaptation to its unique human host and louse vector.

## Treatment

### Pediculosis

Pediculosis can be treated with insecticides, i.e., treating all clothing with 10% DDT, 1% malathion, or 1% permethrin dust (*1*). Because body lice live in clothing, lay their eggs in clothing, and only visit human skin to feed, the patient's body does not need to be deloused. Boiling infested clothes is also efficient (*1*). Changing a person's clothing, including underwear, is the simplest method for delousing, but it is, however, not always practical (*1*). Recently, oral agents have been evaluated, and ivermectin has been efficient in delousing homeless persons in shelters, without other measures (*39*). Bedding at shelters is a major source of infestation and should be treated with insecticides or by boiling the sheets.

### Antimicrobial Drug Susceptibility of *B. quintana*

Evaluation of susceptibilities to antimicrobial drugs has been performed in both axenic media and cell culture. Bacteria of the genus *Bartonella* are susceptible to a wide range of agents, including penicillins, cephalosporins, aminoglycosides, chloramphenicol, tetracyclines, macrolides, rifampin, fluoroquinolones, and cotrimoxazole (*40*). However, only aminoglycosides have a bactericidal effect. MICs correlate poorly with the in vivo efficacies of antimicrobial drugs in patients with *B. quintana* infection, and this discrepancy may be explained by the lack of bactericidal effect of most compounds and by sequestration of the bacterium in erythrocytes (*40*).

### Chronic Bacteremia

In 2003, a randomized trial showed that doxycycline in combination with gentamicin was effective in treating chronic bacteremia. Treatment was given as follows: doxycycline 200 mg/day in 1 oral daily dose for 28 days combined with gentamicin 3 mg/kg/day in 1 intravenous daily dose for 14 days (*40*). This treatment is efficient at the individual level, but *B. quintana* remains endemic in the homeless persons in shelters in Marseilles, France, although bacteremic persons have been treated for 4 years (*17*). Considering the poor adherence of homeless persons to medical care and treatment, a 1-month treatment, including 14 days of intravenous treatment, is difficult to manage in this population. Shorter durations should be evaluated in the future.

### Endocarditis

The efficiency of antimicrobial drugs to treat *Bartonella* endocarditis has recently been evaluated (*25**,**40*). Patients receiving an aminoglycoside are more likely to fully recover (p = 0.02), and those treated with aminoglycosides for >14 days are more likely to survive than those who undergo therapy of shorter duration (p = 0.02) (*25*). The recommended treatment for *B. quintana* endocarditis is as follows: doxycycline 100 mg 2×/day orally for 6 weeks in combination with gentamicin 3 mg/kg /day in 1 intravenous daily dose for 14 days (*40*).

### Bacillary Angiomatosis

Drug treatment of bacillary angiomatosis has not been studied systematically to date, but erythromycin is reported to be efficient and is currently the firstline agent of choice (*40*). Doxycycline has also been recommended, and successful treatment has been reported with ceftriaxone or fluoroquinolones, but treatment with ciprofloxacin was unsuccessful (*40*). The recommended treatment for bacillary angiomatosis is erythromycin 500 mg 4×/day orally for 3 months. In patients with a contraindication to macrolides, doxycycline 100 mg 2×/day orally for 3 months should be considered (*40*). The dramatic efficiency of erythromycin in bacillary angiomatosis is linked to its antiangiogenic effect, rather than to its antimicrobial effect (*15*).

## Conclusion

*B. quintana* infection has long been disconcerting for physicians and researchers. The uncommon quintan fever has been a subject of medical curiosity since 1915, and its clinical course and pathologic features have been understood only after numerous experimental infections in volunteers. The clinical spectrum of *B. quintana* infection includes various manifestations such as bacillary angiomatosis and endocarditis. Treatment of these infections has also been debated and has recently been codified. To date, some points still remain to be elucidated. *B. quintana* was thought to be strictly a human pathogen but was recently detected in cat fleas and monkey fleas, which reopens the debate on the existence of an animal reservoir. The availability of the complete genome sequence of *B. quintana* allows sequence-based typing, which has created convenient tools for molecular epidemiology that are necessary to determine the natural history of *B. quintana* infection.

## Appendix

**Table A1:** Available primers for *Bartonella* spp. Amplification

Microorganism	Gene	Direction*	Primer	Sequence
Bartonella clarridgeiae	ribC	F	PBC5	TACATAACGAGCCAATT
B. clarridgeiae	ribC	R	PBC15	TAGCTTTAGAACAATATGGT
B. henselae	ribC	F	PBH-L1	GATATCGGTTGTGTTGAAGA
B. henselae	ribC	R	PBH-R1	AATAAAAGGTATAAAACGCT
B. bacilliformis	ribC	F	PBH-L1	GATATCGGTTGTGTTGAAGA
B. bacilliformis	ribC	R	PBB-R1	AAAGGCGCTAACTGTTC
B. quintana	ribC	F	PBH-L1	GATATCGGTTGTGTTGAAGA
B. quintana	ribC	R	PBQ-R1	AAAGGGCGTGAATTTTG
All Bartonella	ribC	F	PBH3	CCAAGTGCTACATAACCATC
All Bartonella	ribC	R	PBH4	CGGGTTGTTATTGCTCTTAC
All (except B. bacilliformis)	16s-23s spacer	F	16SF	AGAGGCAGGCAACCACGGTA
All (except B. bacilliformis)	16s-23s spacer	R	23S1	GCCAAGGCATCCACC
All Bartonella	16s-23s spacer	F	QHVE1	TTCAGATGATGATCCCAA
All Bartonella	16s-23s spacer	R	QHVE2	TTGGGATCATCATCTGAA
All Bartonella	16s-23s spacer	F	QHVE3	GATATATTCAGACATGTT
All Bartonella	16s-23s spacer	R	QHVE4	AACATGTCTGAATATATC
B. bacilliformis	16s-23s spacer	F	BABF	CTGGATCACCTCCTTTCTAA
B. bacilliformis	16s-23s spacer	R	BABR	ATGCCCTTAAGACACTTGAT
B. quintana	16s-23s spacer	F	BQF	CTCCACCATTTTAGGTCATC
B. quintana	16s-23s spacer	R	BQR	GGTTTTGAGAATTCCCTTGC
All Bartonella	16s-23s spacer	F	16s1	(C/T)CTTCGTTTCTCTTTCTTCA
All Bartonella	16s-23s spacer	R	16s2	GGATAAACCGGAAAACCTTC
All Bartonella	16s-23s spacer	F	16s3	(C/T)CTTCGTTTCTCTTTCTTCA
All Bartonella	16s-23s spacer	R	16s4	AACCAACTGAGCTACAAGCC
All Bartonella	16s-23s spacer	F	16s5	CTCTTTCTTCAGATGATGATCC
All Bartonella	16s-23s spacer	R	16s6	AACCAACTGAGCTACAAGCCCT
All Bartonella	ribC	F	BARTON-1F	TAACCGATATTGGTTGTGTTGAAG
All Bartonella	ribC	R	BARTON-2R	TAAAGCTAGAAAGTCTGGCAACATAACG
All Bartonella	16s	S	probe	ATTTGGTTGGGCACTCTAGGGG
All Bartonella	16s		Rp2a	ACGGCTACCTTGTTAGGACTT
All Bartonella	16s	F	357f	TACGGGAGGCAGCAG
All Bartonella	16s	R	357ra	CTGCTGCCTCCCGTA
All Bartonella	16s	F	536F	CAGCAGCCGCGGTAATAC
All Bartonella	16s	R	536R	GTATTACCGCGGCTGCTG
All Bartonella	16s	F	800F	ATTAGATACCCTGGTAG
All Bartonella	16s	R	800R	CTACCAGGGTATCTAAT
All Bartonella	16s	F	1050F	TGTCGTCAGCTCGTG
All Bartonella	16s	R	1050R	CACGAGCTGACGACA
All Bartonella	35KD	F	35KD1fa	GTCGCTAAAGGCTGATGA
All Bartonella	35KD	R	35KD2ra	GACTGATATCGTGCGTGTG
All Bartonella	35KD	F	35KDs1f	GGTACGACGACAGTAATTGTT
All Bartonella	35KD	R	35KDs2r	GATTTAAGAGATACCAACCA
All Bartonella	PAP	F	PAP1fa	CTTTAATGACGACTTCTGTT
All Bartonella	PAP	R	PAP4ra	CCGAAATCTGAGTAACGGTA
All Bartonella	PAP	R	PAP2r	CCCTAAATGTTTCAAGTTCA
All Bartonella	PAP	F	PAP3f	GCTGACAGAGAAGACGCAA
All Bartonella	groEL	F	BbHs233.p	CGTGAAGTTGCCTCAAAAACC
All Bartonella	groEL	R	BbHs1630.n	AATCCATTCCGCCCATTC
All Bartonella	rpoB	F	QVE1	TTCAGATGATGATCCCAAGC
All Bartonella	rpoB	R	QVE3	AACATGTCTGAATATATCTTC
All Bartonella			Bfp1	ATTAATCTGCAYCGGCCAGA
All Bartonella			Bfp2	ACVGADACACGAATAACACC
All Bartonella			Bfs3	TTACAAAAATCYGTTGATAC
All Bartonella			Bfs4	GTATCAACRGATTTTTGTAA
All Bartonella	ftsZ	F	BaftsZF	GCTAATCGTATTCGCGAAGAA
All Bartonella	ftsZ	R	BaftsZR	GCTGGTATTTCCAAYTGATCT
B. henselae, B. clarridgeiae	ftsZ		BhftsZ 1393.n	GCGAACTACGGCTTACTTGC
B. henselae, B. clarridgeiae	ftsZ		Bh ftsZ 1247.p	CGGTTGGAGAGCAGTTTCGTC
B. quintana	ftsZ	F	Bq ftsZseqF	GCACATATTCTTGATGAGAT
B. quintana	ftsZ	R	Bq ftsZseqR	CCCCTATCATCTCATCAAG
B. bacilliformis	ftsZ	F	Bb ftsZseqF	GCGCATGTTCTTAGTGAAAT
B. bacilliformis	ftsZ	R	Bb ftsZseqR	CCTGTATACGTGATGCATTT
All Bartonella	ftsZ		FTS1p	GCCTTCTCATCCTCAACTT
All Bartonella	ftsZ		FTS2p	CAGCCTCTTCACGATGTG
All Bartonella	pap31		PAPn1	TTCTAGGAGTTGAAACCGAT
All Bartonella	pap31		PAPn2	GAAACACCACCAGCAACATA
All Bartonella	pap31		PAPns2	GCACCAGACCATTTTTCCTT
All Bartonella	pap31		PAPns1	CAGAGAAGACGCAAAAACCT
All Bartonella	groEL		HSPps1	CAGAAGTTGAAGTGAAAGAAAA
All Bartonella	groEL		HSPps2	GCNGCTTCTTCACCNGCATT
All Bartonella	groEL		HSPps4	GCTGGNGGTGTTGCNGTTA
All Bartonella	groEL		HSPps3	GCTGTNGAAGANGGNATTGT

*F, forward; R, reverse.
